# Correction: Evidence for Distinct Coastal and Offshore Communities of Bottlenose Dolphins in the North East Atlantic

**DOI:** 10.1371/journal.pone.0128259

**Published:** 2015-05-08

**Authors:** The PLOS ONE Staff

## Notice of Republication

This article was republished on April 27, 2015, to correct the distorted Fig 1 and the misspelled species name in the Abstract: "*Tursiops truncatus*" was incorrectly changed to "*Tursiops truncates*." The publisher apologizes for the errors. Please download this article again to view the correct version. The originally published, uncorrected article and the republished, corrected article are provided here for reference.

## Supporting Information

S1 File Originally published, uncorrected article. (PDF)

S2 File Republished, corrected article. (PDF)
