# Supplementary material for: Correction: Evidence for Distinct Coastal and Offshore Communities of Bottlenose Dolphins in the North East Atlantic
Source: PLoS One. 2015 May 8;10(5):e0128259. doi: 10.1371/journal.pone.0128259 (PMC4425471; doi:10.1371/journal.pone.0128259)
Supplement: S2 File — (PDF) [file pone.0128259.s002.pdf]

PLOS ONE

RESEARCH ARTICLE

Evidence for Distinct Coastal and Offshore Communities of Bottlenose Dolphins in the North East Atlantic

Machiel G. Oudejans[1,2]*, **Fleur Visser**[2,3], **Anneli Englund**[4], **Emer Rogan**[4], **Simon N. Ingram**[4,5]

1 Dúlra Research, Heiloo, The Netherlands, **2** Kelp Marine Research, Hoorn, The Netherlands, **3** Behavioural Biology Group, Leiden University, Leiden, The Netherlands, **4** School of Biological, Earth and Environmental Sciences, University College Cork, Ireland, **5** Marine Vertebrate Research Group, School of Marine Science and Engineering, Plymouth University, Drake Circus, Plymouth, United Kingdom

* machiel.oudejans@gmail.com

CrossMark
click for updates

OPEN ACCESS

Citation: Oudejans MG, Visser F, Englund A, Rogan E, Ingram SN (2015) Evidence for Distinct Coastal and Offshore Communities of Bottlenose Dolphins in the North East Atlantic. PLoS ONE 10(4): e0122668. doi:10.1371/journal.pone.0122668

Academic Editor: Ruth H. Carmichael, Dauphin Island Sea Lab; University of South Alabama, UNITED STATES

Received: February 12, 2014

Accepted: February 24, 2015

Published: April 8, 2015

Copyright: © 2015 Oudejans et al. This is an open access article distributed under the terms of the Creative Commons Attribution License, which permits unrestricted use, distribution, and reproduction in any medium, provided the original author and source are credited.

Funding: Data collection was funded from a number of different sources and the authors are grateful to Science Foundation Ireland, The National Parks and Wildlife Service, The Heritage Council and the Fiontar Bellacorick Fund for funding. The funders had no role in study design, data collection and analysis, decision to publish, or preparation of the manuscript. Dúlra Research and Kelp Marine Research provided support in the form of salaries for authors MGO and FV, but did not have any additional role in the study design, data collection and analysis, decision to publish, or preparation of the manuscript. The specific

Abstract

Bottlenose dolphin stock structure in the northeast Atlantic remains poorly understood. However, fine scale photo-id data have shown that populations can comprise multiple overlapping social communities. These social communities form structural elements of bottlenose dolphin (*Tursiops truncatus*) populations, reflecting specific ecological and behavioural adaptations to local habitats. We investigated the social structure of bottlenose dolphins in the waters of northwest Ireland and present evidence for distinct inshore and offshore social communities. Individuals of the inshore community had a coastal distribution restricted to waters within 3 km from shore. These animals exhibited a cohesive, fission-fusion social organisation, with repeated resightings within the research area, within a larger coastal home range. The offshore community comprised one or more distinct groups, found significantly further offshore (>4 km) than the inshore animals. In addition, dorsal fin scarring patterns differed significantly between inshore and offshore communities with individuals of the offshore community having more distinctly marked dorsal fins. Specifically, almost half of the individuals in the offshore community (48%) had characteristic stereotyped damage to the tip of the dorsal fin, rarely recorded in the inshore community (7%). We propose that this characteristic is likely due to interactions with pelagic fisheries. Social segregation and scarring differences found here indicate that the distinct communities are likely to be spatially and behaviourally segregated. Together with recent genetic evidence of distinct offshore and coastal population structures, this provides evidence for bottlenose dolphin inshore/offshore community differentiation in the northeast Atlantic. We recommend that social communities should be considered as fundamental units for the management and conservation of bottlenose dolphins and their habitat specialisations.

roles of these authors are articulated in the 'author contributions' section.

Competing Interests: The authors have the following interests. Machiel G. Oudejans is affiliated with Dúlra Research and Kelp Marine Research and Fleur Visser with Kelp Marine Research. There are no patents, products in development or marketed products to declare. This does not alter the authors' adherence to all the PLOS ONE policies on sharing data and materials, as detailed online in the guide for authors.

Introduction

Bottlenose dolphins (*Tursiops truncatus*) inhabit a wide range of habitats throughout their worldwide distribution [1]. The ecological plasticity of this highly mobile species facilitates interaction and gene flow over large distances [2–4]. However, bottlenose dolphin populations commonly consist of distinct social communities that display fine-scale behavioural differentiation, resulting from localised adaptations on small spatial scales [5–8] resulting in fine scale genetic structuring [9,10]. A community can be defined as a set of individuals that is behaviourally self-contained and within which most individuals interact with most others [11], and is formed when a subgroup of a population develops group-specific adaptations or behavioural specialisations [8,12–14], or forms an isolated social unit [15]. In restricted coastal habitats, bottlenose dolphin communities are generally dominated by small but highly fluid schools, constricted movement patterns and high site-fidelity [16–18]. In contrast, bottlenose dolphins inhabiting open, exposed habitat occur in large groups, with low site-fidelity and extensive movement patterns [19,20]. In the northwest Atlantic and the northeast Pacific, inshore and offshore ecotypes of bottlenose dolphins have been identified based on morphological [21,22], ecological [23,25] and genetic [9,26,27] differences.

In Ireland, bottlenose dolphins are found in estuarine, coastal, continental shelf and oceanic waters [28–31]. To date, at least three genetically distinct populations have been identified [10]: a resident population inhabiting the Shannon estuary, a population inhabiting the coastal waters of western Ireland and a population identified genetically from stranding records of unknown origin, possibly representing an oceanic population [10]. Individuals of the coastal population have been documented making large-scale movements, around Ireland [30], and between Atlantic coastal waters and the North Sea [32]. It remains uncertain whether these large scale movements represent individual or population-wide ranging patterns. Despite their use of adjacent habitats, there is currently no evidence of interactions between bottlenose dolphins inhabiting the Shannon estuary and those using other Irish areas and offshore waters [30] and these communities appear to represent different breeding populations.

Here, we investigate the community structure of bottlenose dolphins around northwest Ireland in coastal and offshore habitats. Using social network analysis, encounter locations and fin scarring patterns, we test whether bottlenose dolphins using coastal and continental shelf waters belong to a single or multiple communities.

Materials and Methods

Study area

Vessel-based surveys were conducted using a variety of small boats in offshore and inshore areas off northwest Ireland in order to collect photographic identification data of encountered bottlenose dolphins for individual identification (Fig. 1). Inshore surveys were conducted in two coastal areas, referred to as 'Mayo' around the Mullet Peninsula Co. Mayo (N 54,10° E 10,06°) and 'Connemara' around northwest Connemara, Co. Galway (N 53,60° E 10,10°). Three surveys covered oceanic waters (>200m water depth) near the continental shelf edge, northwest of the Mullet peninsula, Co. Mayo.

Survey observations

All surveys were conducted between March and October 2008–2012 during fair weather conditions with sea states <4 on the Beaufort scale. For each encounter, the geographical location was recorded at first sighting of the group. A group was defined as all dolphins within a 100 m

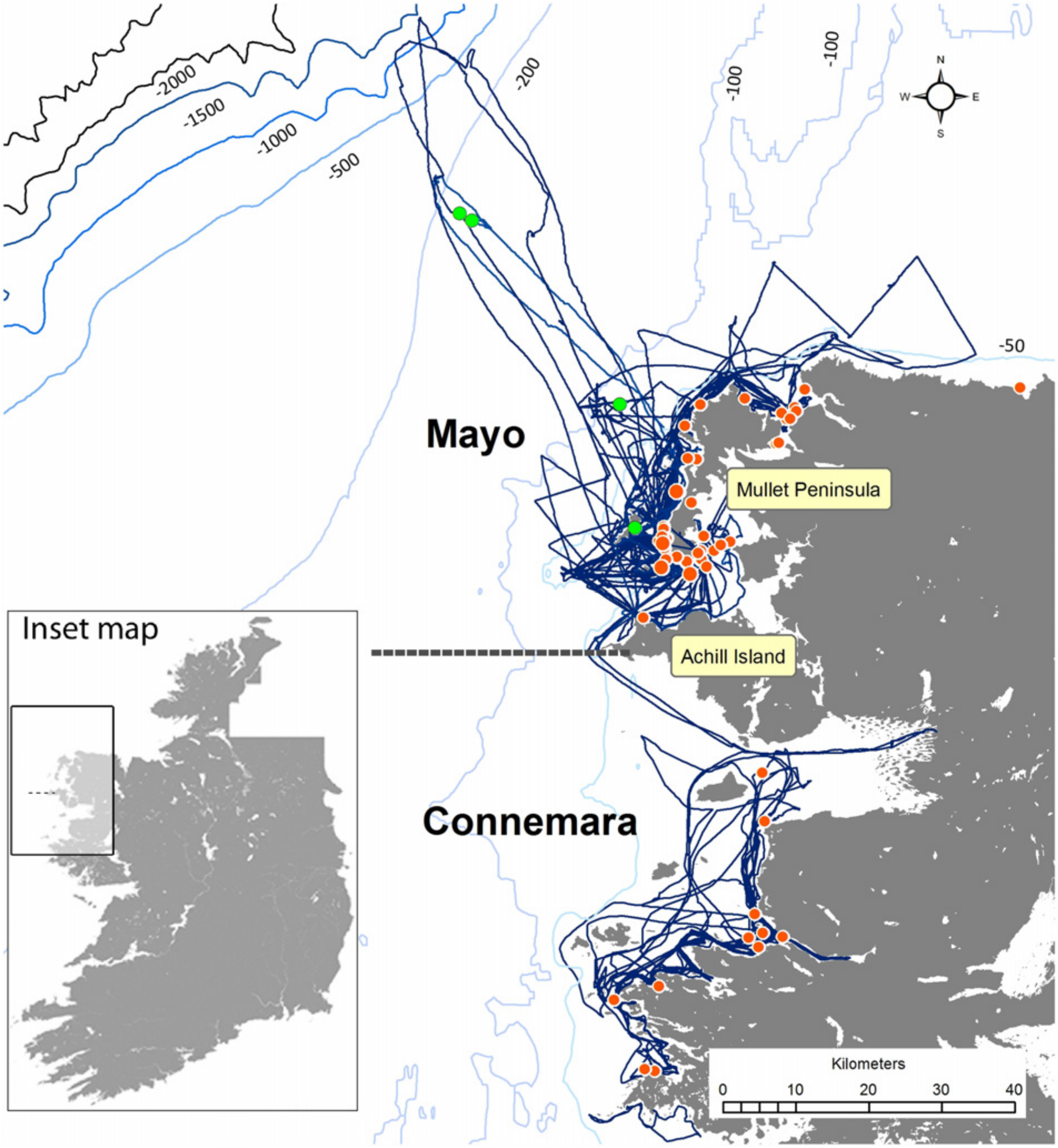

Fig 1. Survey effort and sighting locations of bottlenose dolphin group encounters. The black lines indicate survey effort in the two research areas off northwest Ireland, Mayo and Connemara. Sighting locations of bottlenose dolphin groups in inshore waters are indicated by red circles, encounters in offshore waters are indicated by green circles. The geological map of Ireland has been reproduced under an open-access license from the DECRN, and the Intellectual Property Right and Copyright of the Geological Service Ireland. The bathymetry is reproduced from GEBCO, and extracted from the GEBCO Digital Atlas published by the British Oceanographic Data Centre on behalf of the IOC and IHO, 2003.

doi:10.1371/journal.pone.0122668.g001

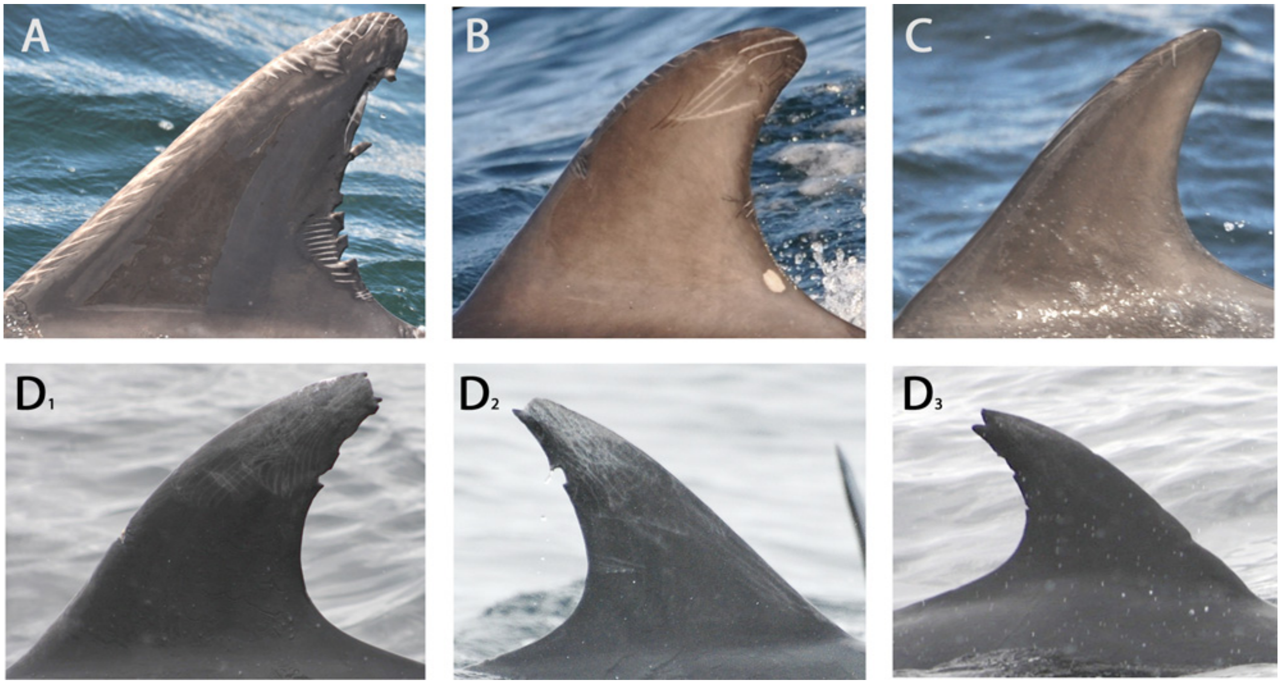

Fig 2. Grades of severity of dorsal fin scarification. Examples of the mark severity grades of dorsal fin scarification as applied in this study. (A) Permanently marked, (B) Temporarily marked, (C) Superficially marked, (D1-3) Permanently marked with damage to the tip of the dorsal fin. Pictures represent images cropped using Irfanview software.

doi:10.1371/journal.pone.0122668.g002

radius of each other, showing coordinated movement patterns and behaviour during the encounter [33].

Photo identification of individuals

Dedicated effort was made to photograph all individuals in the group using digital DSLR cameras with telephoto lenses. We used standard photo-identification techniques to identify individual dolphins [34,35]. Photograph quality was classified based on focus, angle, light and distance to the subject [36]. Each individual was assigned one of three marking grades based on the severity of scarring of the dorsal fin: permanently, temporarily or superficially marked (mark severity; Fig. 2A-C). Permanently marked animals had deep and/or large scars and cuts on the dorsal fin that enabled individual identification over the duration of the project [36,37]. Temporarily marked animals had small cuts, light scars and/or tooth rakes on the dorsal fin, which may fade and heal within a single year [36]. Superficially marked animals had only superficial rakes and lesions on the dorsal fin. Photographs from each encounter were matched against a catalogue consisting of the best left and right photos of dolphins identified during previous encounters, each assigned a unique identification number. If photos of an individual did not match with animals in the catalogue, a new entry was added to the catalogue and a new unique identification number assigned to the individual.

Analysis of social structure

We used identification data in a network analysis to investigate social structure, and constructed a social network diagram [38]. Individual dolphins were considered associated if they were identified within the same group (gambit of the group [39]). Only permanently marked individuals were included in the network analysis enabling matching between years and

Table 1. Survey effort and photo identification analysis.

Year	2008	2009	2010	2011	2012	Total
No. of surveys	33	43	24	5	12	117
No. of group encounters	12	18	12	14	4	60
No. of identifications	105	422	374	310	97	1308
No. of unique dolphins identified	84	122	172	133	61	286
% of the catalogue identified	29%	43%	60%	47%	21%	100%

The number of surveys, group encounters, total number of dolphins recorded each year, the number of individuals in the catalogue identified per year, and the percentage of the catalogued individuals identified per year.

doi:10.1371/journal.pone.0122668.t001

research areas. Since the aim of this study was to analyse social interactions at the level of the community, and not between dyads, we included all individuals regardless of sighting frequency. We constructed a sighting database to analyse social affiliation patterns and calculate the association strength between permanently marked individuals by computing pair-wise association indices, using the half-weight association index (HWI) [40]. The HWI is defined as: HWI = X / (X + 0.5 ($Y_a + Y_b$)), where *X* is the number of groups in which individual *a* and individual *b* were seen together, Y_a is the number of groups in which individual *a* was sighted but not individual *b*, and Y_b is the number of groups in which individual *b* was sighted but not individual *a*. Values of the HWI range from 0 (never associated) to 1 (always associated) [39]. The HWI is frequently used in social studies of cetaceans as it reduces bias due to incomplete identification within encounters [40]. The social network analysis was conducted using SOCPROG version 2.4 [41]. We used the pair-wise association indices to visualize the social network analysis in Netdraw 2.118 [42].

As a second step, we investigated the community membership of the temporarily and superficially marked individuals. This was done by scoring the network membership of the permanently marked individuals with which it was associated. A temporarily/superficially marked individual was assigned to a network if all its permanently marked associates belonged to that network.

Ethical statement

The National Parks and Wildlife Service, a division of the Irish governmental Department of Arts, Heritage and the Gaeltacht is responsible for the protection and management of Irish wildlife, and the designation and protection of Special Areas of Conservation. No specific permission or permit was required for the fieldwork/data collection, which was conducted in Irish coastal waters in county Mayo (N 54,10° E 10,06°), and in Connemara, county Galway (N 53,60° E 10,10°). Shore-based effort was conducted from public beaches and roads, and did not access private land. All wildlife in Ireland, including the bottlenose dolphin, are protected under the Wildlife Act, 1976. In addition, the bottlenose dolphin is listed under Annex II of the EC Habitats Directive. The current status of the species is Least Concern (IUCN Red List 2012). The study did not involve the handling or management of dolphins.

All research effort complied with the Marine Notice No. 15 of 2005 "*Guidelines For Correct Procedures When Encountering Whales And Dolphins In Irish Coastal Waters*", published by the Department of Communication, Marine and Natural Resources [43].

During data collection at sea the following protocols were followed to minimize disturbance: maintain minimal vessel speed and minimal number of directional changes of the vessel

course. The behaviour of dolphins around the survey vessel was constantly monitored. If strong behavioural responses were observed (e.g., continued loud exhalations or tail slaps), the survey protocol was suspended and the encounter was terminated.

Results

Survey effort

A total of 117 surveys were conducted between 2008 and 2012 (Fig. 1, Table 1). Survey effort was not distributed uniformly between the two study areas, with higher survey effort recorded in Mayo. More survey effort was conducted in coastal waters <10km from shore. Offshore surveys covering continental shelf edge waters >200m depth were limited to three survey days in Mayo, while no offshore surveys were conducted from Connemara.

Bottlenose dolphin identifications

In total we had 51 encounters with bottlenose dolphin groups in Mayo and 9 encounters in Connemara (Table 1). The number of individuals identified per group ranged from 1–74 individuals, with a mean (±SD) of 21.8 ± 19.3. Group size did not differ significantly between the two research areas (Mann Whitney U test, U = 204, N = 60, P = 0.60).

During the study, a total of 286 individual dolphins were catalogued and the number of sightings of individual dolphins ranged from 1 to 23 (Fig. 3). In total, 163 individuals (57%) were recorded during two or more encounters of which 139 individuals (49%) were recorded in multiple years (Fig. 3, Table 1). More individuals were recorded in Mayo than in Connemara, 279 vs. 86 individuals. 79 individuals were sighted in both coastal areas representing 28% and 92% of all dolphins identified in Mayo and Connemara respectively. 152 individuals (53%)

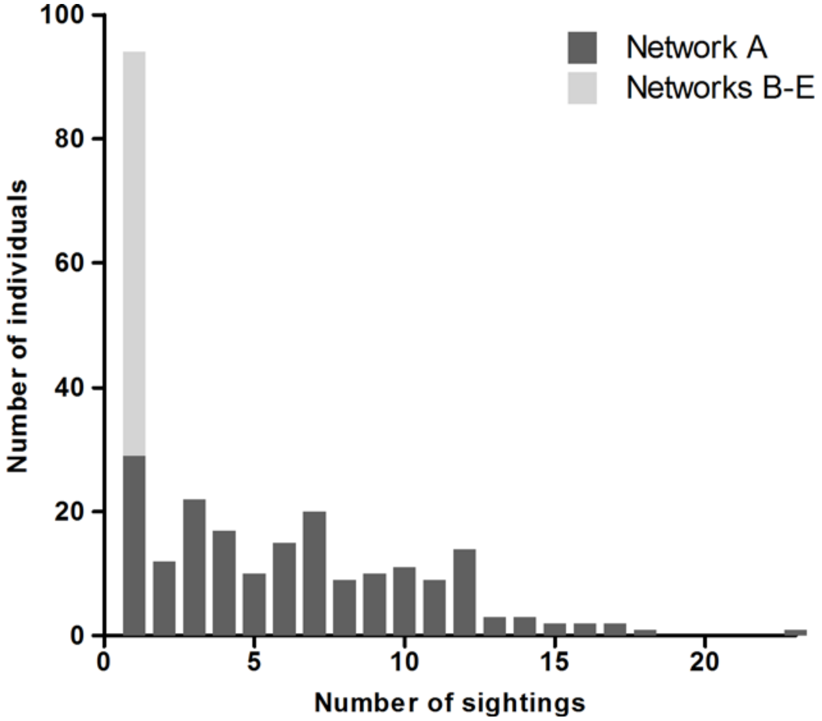

Fig 3. The number of sightings of individuals comprising network A and network B-E.

doi:10.1371/journal.pone.0122668.g003

Table 2. Statistic descriptive of mark severity, dorsal tip damage and sighting record for network A and network B-E.

Network	A	B–E	Total
Superficial	46	6	52
Temporal	74	8	82
Permanent	72	80	152
Total	192	94	286
% dorsal tip scarring	7%	48%	20%
% sighted once	15%	100%	43%
% sighted >1	85%	0%	57%

The number of individual dolphins for each mark severity, the percentage of dolphins with scarring to the tip of the dorsal fin, individuals sighted once, and resighted, for network A (inshore) and network B–E (offshore) communities.

doi:10.1371/journal.pone.0122668.t002

of the dolphins identified had permanently marked dorsal fins, whereas 29% and 19% were temporarily or superficially marked, respectively (Table 2).

Social networks

The network analysis of the association indices of 152 permanently marked individuals identified five clusters: one large network (A) and four smaller networks (B-E) (Fig. 4). Network A comprised 72 individuals, identified during 56 encounters (mean group size (±SD) = 10.4 ± 11.1). This network incorporated all 42 permanently marked individuals recorded in

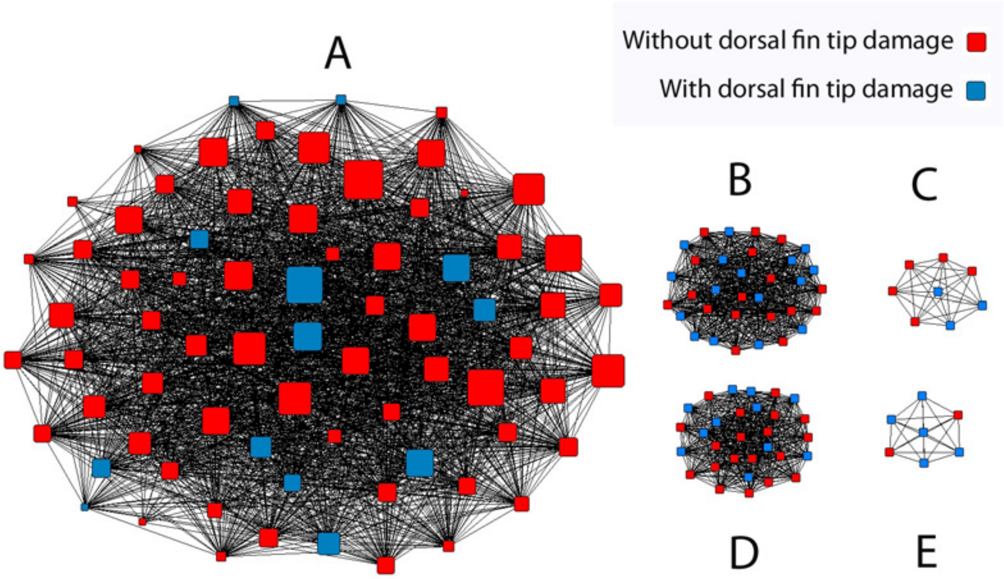

Fig 4. Social network analysis: sociogram of permanently marked individuals. Five social clusters (A-E), as identified by the social network analysis of associations of permanently marked bottlenose dolphins. Each symbol represents one individual, with the size of the symbols corresponding to the number of sightings of each individual (range: 1–18 sightings per individual). Blue squares represent individuals with typical scarring to the tip of the dorsal fin. Individuals without dorsal fin tip damage are represented by red squares. Black lines represent associations between individuals. The network composition has been manually adjusted to enhance the visualization of the separate clusters.

doi:10.1371/journal.pone.0122668.g004

Connemara and Mayo, with an additional 30 individuals recorded in Mayo. Almost all individuals in network A were sighted more than once (96%, N = 69), and over half the individuals (56%) were recorded in 3 or more years during the 5 year study (Table 2). In total, 83 permanently marked individuals were sighted only once, of which only three individuals were assigned to network A. The four smaller networks comprised 80 permanently marked individuals (network B-E). These individuals were recorded in Mayo during 4 separate group encounters (group size = 8, 9, 30 and 33). None of these individuals were resighted and thus did not share any associations with other groups, or with network A (Fig. 4). This strongly contrasted with the 96% of network A individuals that were sighted at least twice. The majority of the temporarily and superficially scarred individuals, 74 out of 82 and 46 out of 52, respectively, were assigned to network A, based on shared associations with permanently marked individuals comprising this network (Table 2).

Dorsal fin marking severity

The majority of individuals, 152 (53%), identified had permanently marked dorsal fins, whereas 29% and 19% were temporarily or superficially marked (Table 2). The proportion of permanently marked individuals differed significantly between the five network clusters (Chi square test, $\chi = 60.6$, N = 286, df = 4, $P < 0.001$). Networks B-E predominantly consisted of permanently marked individuals (85%; N = 80), whereas less than half of the individuals of network A (38%; N = 72), were permanently marked. Pair-wise Chi square tests between the networks identified differences in proportion of permanently marked individuals between the small networks and the large network A individuals, but not among any of the smaller networks (Table 3).

Additionally, photographic analysis identified a characteristic type of damage to the tip of the dorsal fin (Fig. 2D1-3) which was observed in a significantly higher proportion of individuals belonging to networks B-E, than network A, (48% and 7% of the dolphins in networks B-E and network A respectively) (Chi square test, $\chi = 23.4$, N = 152, df = 1, $P < 0.01$; Table 2).

Spatial distribution of dolphin groups

Encounters with dolphin groups ranged between 50 m and 40.5 km from shore (Figs. 1 & 5). Encounters with animals from network A were significantly closer to shore (mean distance ± SD = 0.61 ± 0.61 km), than encounters with animals belonging to network B-E (23.2 ± 19.0 km; Mann-Whitney U: U = 224, N = 60, $P < 0.05$; Figs. 1 & 5). All 56 encounters with groups comprised of network A dolphins were within 3 km from shore. In contrast the four groups representing networks B-E were recorded between 4.6 and 40.5 km from the mainland.

Table 3. Chi square pair-wise comparison of the proportion of permanently marked individuals for social networks A-E.

Network	A (72–120)	B (33–9)	C (8–3)	D (30–1)	E (9–1)
B	**χ = 23.5 P<0.005**				
C	χ = 5.4 P = 0.201	χ = 0.17 P = 0.680			
D	**χ = 37.8 P<0.005**	χ = 4.9 p = 0.253	χ = 5.4 p = 0.196		
E	**χ = 10.9 P<0.005**	χ = 0.67 P = 0.410	χ = 1.0 P = 0.314	χ = 0.75 P = 0.387	-

Chi square pair-wise comparison between the networks (A-E) for the proportion of permanent marked individuals. Significant differences between networks are shown in bold for (P-value 0.0050 (0.05/9). Degrees of freedom = 1 for all comparisons, between brackets (number of permanent—non-permanent marked individuals).

doi:10.1371/journal.pone.0122668.t003

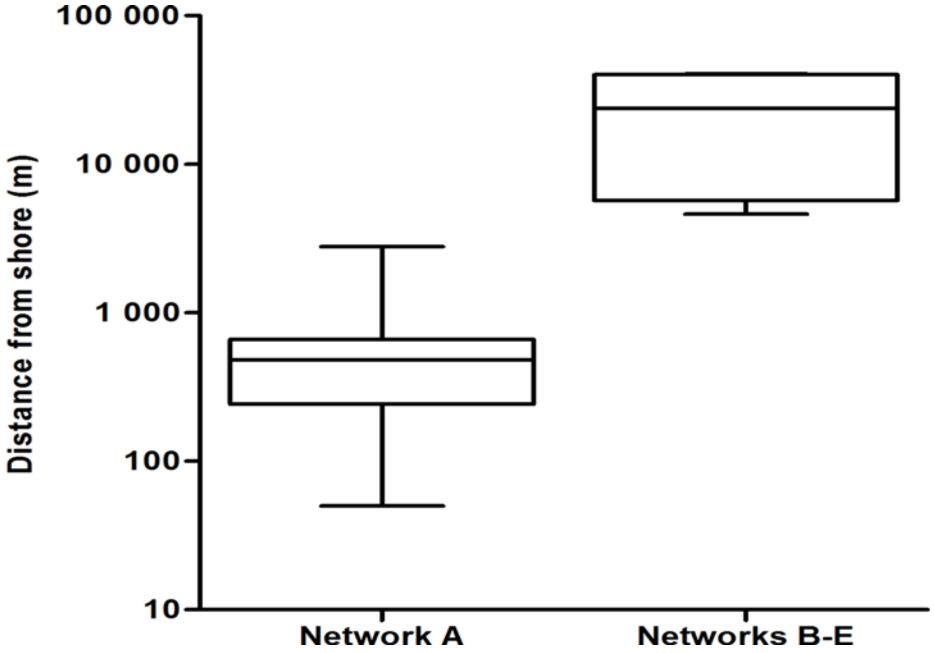

Fig 5. Distance from shore. The distance from shore for bottlenose dolphin groups that comprised network A (left) and networks B-E (right). The boxplot displays the mean and the 1st and 3rd quartile of the distance from shore. Whiskers indicate one standard deviation from the mean. Note the logarithmic scale of the Y-axis.

doi:10.1371/journal.pone.0122668.g005

Discussion

Our results indicate that bottlenose dolphins in the waters off the northwest Irish coast are segregated into one distinct inshore and one or multiple offshore social communities. Inshore community group encounters were restricted to coastal waters within 3 km from shore. Offshore community groups were found significantly further from shore (4.5–41km). Dolphins of the two communities also differed significantly in their degree of dorsal fin damage. Significantly more individuals in the offshore community were heavily scarred. They were also typified by characteristic dorsal fin tip damage, which was rarely observed in dolphins in the inshore community. Furthermore, inshore community dolphins showed inter-annual site fidelity to the coastal zone sites and the distribution of their encounters fits a linear coastal distribution consistent with the genetic haplotype patterns [10]. In contrast, dolphins in offshore waters were never observed more than once. This may reflect limited survey effort and/or a relatively low number of sightings in the offshore area. However, all of the recorded inshore groups formed a single network cluster and thus interacted, while none of the individuals in these groups showed an association with any of the offshore dolphins. These differences provide evidence that these inshore and offshore social communities are also spatially and behaviourally segregated, which may extend to genetic differentiation, but requires additional study.

The inshore community

The social network analysis showed that the bottlenose dolphins encountered in inshore waters of Mayo and Connemara form a single cohesive social community whose movements appear to be restricted to a 3 km strip of coastal habitat. Dolphins belonging to this community display

a fission-fusion interaction typical for coastal bottlenose dolphin communities around the world, whereby individuals associate and dis-associate at varying time-scales [44]. These findings are consistent with genetic evidence showing that individuals in the Connemara-Mayo coastal waters belong to a single breeding population, which is genetically distinct from the Shannon estuary population, and a third unidentified population identified in Irish waters [10]. Interestingly, inshore community groups were mostly observed very close to the coast, often within a few hundred meters from shore. Distribution patterns confined to this narrow band are further supported by the movement of groups which were tracked for periods up to 8 hours [30; M. Oudejans, unpublished data], and indicate that this community utilises the shallow, intertidal areas along the beaches and rocky areas which typify this coastal region.

The offshore community

The existence of an oceanic population was suggested by Mirimin and colleagues [10], based on high levels of genetic diversity in samples of stranded dolphins, which were genetically distinct from animals biopsied in Irish coastal waters. We recorded four groups that shared no associations with the inshore community. Individuals in these groups were distinguished by their offshore distribution (> 4 km), low sighting rates and high degree of dorsal fin damage. While based on a limited number of encounters, low re-sighting rates would be consistent with a larger offshore population with animals displaying a more extensive ranging behaviour, and wide scale movement patterns [20,23]. Hereby, our findings support evidence for a distinct offshore population, and show this population to be socially and ecologically discrete from the coastal population.

Evidence for ecotype differentiation

Noticeable similarities in distribution and sighting frequency exist between the inshore and offshore communities in Ireland, and coastal and offshore ecotypes in the western Atlantic and eastern Pacific, where the coastal ecotype inhabits the inshore waters within 7.5 km and 1 km from shore, respectively [24,45]. Within this narrow band, individual dolphins move extensively but remain close to shore [46]. The offshore ecotype is distributed further offshore, and its distribution is largely associated with the continental shelf edge [23–25]. Here, all inshore community members were recorded within coastal waters, moving between research areas, and likely beyond [30]. Moreover, members of groups encountered in waters further offshore were all seen on single occasions, possibly reflecting a more transient nature of these individuals. The distinct social partition between communities and the parallels in distribution pattern and differences in sighting frequency with northeast Atlantic and northwest Pacific ecotypes suggests that these two Irish communities may also represent two ecotypes, an inshore and an offshore ecotype. Recent genetic studies have identified the presence of inshore and offshore ecotypes on a regional scale in the northeast Atlantic [9]. Here our data focus on a smaller scale, and provide evidence from populations in an understudied area, in which offshore dolphins have not yet been sampled. Hence, further research, including genetic sampling of the two social communities, is needed to determine whether the spatial, social and behavioural segregation has also led to different breeding populations.

Methods involving the use of marking characteristics of the dorsal fin are widely used in identification of individual cetaceans, in particular for the bottlenose dolphin. Yet, few studies have studied differences of variation in markings at a community or population level. Baird and colleagues [47,48] found significant differences in dorsal fin disfigurements resulting from interactions with fisheries between three populations of false killer whales (*Pseudorca crassidens*) in Hawaii. Interestingly, a three-fold difference in injury rate between social clusters of

false killer whales was observed, which suggest social groups have different interaction rates with fisheries [47]. A similar disparity in the proportion of marked individuals was found for transient and resident groups of short-finned pilot whales (*Globicephala macrorhynchus*) off Madeira [49]. Offshore bottlenose dolphins in our study showed a typical type of damage at the tip of the dorsal fin that was rarely recorded in the inshore community. Markings on the dorsal fin result from interactions with conspecifics [50], predators or prey [51] and human activities including boat strikes, fishing gear or propeller wounds [52,53]. Dorsal fin injuries from sharks appear to be relatively uncommon for bottlenose dolphins and are typically jagged shaped [54], unlike the dorsal fin damage observed here (Fig. 2).

While interactions with fisheries provide foraging opportunities, they also present risks of injury and mortality through entanglement in fishing gear. Fishing interactions were found to be the most common cause of injuries to dolphins in Aruban waters [53]. Subsurface video of interactions between bottlenose dolphins and demersal trawl fisheries off western Australia showed dolphins commonly foraging inside, and physically in contact with trawling nets [55]. Such interactions are likely to result in damage to the dorsal fin and other parts of the body, as indicated by the high number of bycatch of ~40 dolphins annually for these fisheries [56]. The observed differences in marking severity and proportion of atypical damage to the tip of the dorsal fin between dolphins in inshore (<3km) and offshore waters, can reflect different habitat related foraging behaviours. Moreover, fisheries interactions may potentially lead to the development of a characteristic scar. The continental shelf edge and oceanic waters west of Ireland are important fishing grounds for the European commercial pelagic and demersal fisheries [57]. Bottlenose dolphins interact with, and are occasionally caught in nets of mid-water trawlers [58], and driftnet fisheries [59]. In contrast, commercial fishing effort is relatively low in coastal waters, and mainly involves small-scale crab and lobster fisheries that use static gear, and gill net fisheries [57]. Accordingly, dolphins in neritic and oceanic waters are more likely to interact with commercial fisheries than dolphins in inshore waters, which will increase the likelihood of damage to the dorsal fin.

Community overlap

We cannot exclude the possibility that inshore and offshore communities have overlapping ranges. Resident inshore dolphins in Galicia, Spain conducted offshore foraging trips [60], and a small number of coastal ecotype bottlenose dolphins off California have been recorded in offshore waters, outside their general home-range [25]. The close proximity of the continental shelf edge, located 75 km from shore in this region, facilitates oceanic species to occasionally venture into coastal waters, as illustrated by occasional sightings of oceanic species in these waters, such as the sei whale (*Balaenoptera borealis*; [61] and long-finned pilot whales (*Globicephala melas*; Oudejans unpublished data). It is possible that offshore animals encountered during this study are a nearshore component of a larger shelf edge and neritic stock. Future studies to evaluate this coastal and offshore region will be important to answer this question.

Conservation and management implications

This study contributes to a growing body of evidence that inshore coastal communities of bottlenose dolphins in European waters should be managed separately from offshore communities. Genetic diversity between coastal and pelagic populations in the Atlantic Ocean indicate that coastal populations originate from historic founder events of pelagic source populations [3,9,27]. Many coastal populations became highly specialized to their environment, mediated by behavioural specializations to local habitat [62]. Community membership of these coastal populations of bottlenose dolphins can be largely stable across multiple generations [16].

Consequently, with coastal ecosystems under increasing anthropogenic pressure, inshore communities with restricted ranges and relatively narrow ecological niches adapted to local habitat, face significant risks [63]. Our study shows that the Mayo-Connemara area likely forms a key part of the home range of the inshore community of bottlenose dolphins in Irish waters. This implies that degradation, or loss of this habitat can have consequences at the population level [64]. In 2013, a significant part of the coastal Mayo and Connemara research area was designated as the West Connaught Coast candidate Special Area of Conservation (cSAC) for the bottlenose dolphin, designated under the EU Habitat Directive 92/42/EEC. The SACs are key elements of the Habitat Directive, which was developed to maintain and restore natural habitats and species of wild fauna and flora at a favourable conservation status. Further research into population status, habitat-use and movement patterns is necessary in order to monitor and maintain the status of this population adequately.

For offshore cetacean populations, injuries through interactions with fisheries form one of the greatest conservation concerns [65]. Here, individuals of the offshore community were distinguished by pronounced scarring and characteristic dorsal fin damage. This suggests frequent fisheries interactions in offshore waters, and may indicate an undetected or underreported threat to bottlenose dolphins in offshore, shelf edge and oceanic habitats. Further genetic studies are required to elucidate if the networks found in this study represent genetically distinct populations, and if the animals seen in offshore waters represent a single population or comprise multiple populations. Identification of genetically and/or socially distinct communities form a fundamental aspect in the successful long-term management and monitoring of bottlenose dolphin communities and populations, allowing for targeted management measures, tailored to the behavioural and habitat specialisations.

Acknowledgments

We thank the researchers, Erris inshore fishermen, volunteers and members of the public who reported sightings and assisted with the collection of identification photographs. In particular Anthony Irwin, Vincent and Simon Sweeney, John Britain, John Mongan, the crews of the *Dúlra na Mara*, Killary Cruises and Scuba Dive West, the CMRC Broadhaven Bay research team, Oliver Ó Cadhla, Dave Suddaby, Brian Mac Suibhne, Cillian and Gemma O'Mongain, Cormac de Roiste, Jackie Hunt, Dan Brown and Will Woodrow. Additionally, the authors would like to thank Steve Shippee and one anonymous reviewer who provided invaluable feedback on the manuscript.

Author Contributions

Conceived and designed the experiments: MGO FV AE SNI ER. Performed the experiments: MGO FV AE SNI ER. Analyzed the data: MGO FV. Contributed reagents/materials/analysis tools: MGO. Wrote the paper: MGO FV AE SNI ER.

References

1. Leatherwood S, Reeves R (1983) The Sierra Club handbook of whales and dolphins. San Francisco. Sierra Club Books. 302 p.
2. Hoelzel AR (1998) Genetic structure of cetacean populations in sympatry, parapatry and mixed assemblages; implications for conservation policy. Journal of Heredity 89: 451–458.
3. Querouil S, Silva MA, Freitas L, Prieto R, Magalhae S, Dinis A, et al. (2007) High gene flow in oceanic bottlenose dolphins (*Tursiops truncatus*) of the North Atlantic. Conservation Genetics 8: 1405–1419.
4. Tezanos-Pinto G, Baker CS, Russell K, Martien K, Baird RW, Hutt A, et al. (2009) A worldwide perspective on the population structure and genetic diversity of bottlenose dolphins (*Tursiops truncatus*) in New Zealand. Journal of Heredity 100: 11–24. doi: 10.1093/jhered/esn039 PMID: 18495650

5. Caldwell M (2001) Social and genetic structure of bottlenose dolphin (*Tursiops truncatus*) in Jacksonville, Florida. PhD Thesis, University of Miami, Miami.
6. Möller LM, Wiszniewski J, Allen SJ, Beheregaray LB (2007) Habitat type promotes rapid and extremely localised genetic differentiation in dolphins. Marine and Freshwater Research 58: 640–648.
7. Ansmann IC, Parra GJ, Lanyon JM, Seddon JM (2012) Fine-scale genetic population structure in a mobile marine mammal: inshore bottlenose dolphins in Moreton Bay, Australia. Molecular Ecology 21: 4472–4485. doi: 10.1111/j.1365-294X.2012.05722.x PMID: 22882348
8. Daura-Jorge FG, Cantor M, Ingram SN, Lusseau D, Simões-Lopes PC (2012) The structure of a bottlenose dolphin society is coupled to a unique foraging cooperation with artisanal fishermen. Biological Letters 8: 702–705.
9. Louis M, Viricel A, Lucas T, Peltier H, Alfonsi E, Berrow S, et al. (2014) Habitat-driven population structure of bottlenose dolphins, *Tursiops truncatus*, in the North-East Atlantic. Molecular Ecology 23(4): 857–874. doi: 10.1111/mec.12653 PMID: 24383934
10. Mirimin L, Miller R, Dillane E, Berrow SD, Ingram SN, Cross TF, Rogan E (2011) Fine-scale population genetic structuring of bottlenose dolphins in Irish coastal waters. Animal Conservation 14: 342–353.
11. Wells R, Scott MD, Irvine AB (1987) The social structure of free-ranging bottlenose dolphins. In: Genoways HH, editor. Current Mammology. New York, Plenum Press. 540 p.
12. Chilvers BL, Corkeron PJ (2001) Trawling and bottlenose dolphins' social structure. Proceedings of the Royal Society of London 268: 1901–1905. PMID: 11564345
13. Wiszniewski J, Allen SJ, Möller LM (2009) Social cohesion in a hierarchically structured embayment population of Indo-Pacific bottlenose dolphins. Animal Behaviour 77: 1449–1457.
14. Mann J, Stanton MA, Patterson EM, Bienenstock EJ, Singh LO (2012) Social networks reveal cultural behaviour in tool-using using dolphins. Nature communications 3: 980 doi: 10.1038/ncomms1983 PMID: 22864573
15. David L, Schneider K, Boisseau OJ, Haase P, Slooten E, Dawson SM (2003) The bottlenose dolphin community of Doubtful Sound features a large proportion of long-lasting associations. Behavioral Ecology and Sociobiology 54: 396–405
16. Wells R (2003) Dolphin social complexity: Lessons from the long-term study and life history. In: De Waal FB, Tyack PL, editors. Animal social complexity, Harvard University Press. pp. 32–56.
17. Ingram SN, Rogan E (2002) Identifying critical areas and habitat preferences of bottlenose dolphins (*Tursiops truncatus*). Marine Ecology Progress Series 244: 247–255.
18. Wiszniewski J, Beheregaray LB, Allen SJ, Möller LM (2010) Environmental and social influences on the genetic structure of bottlenose dolphins (*Tursiops aduncus*) in Southeastern Australia. Conservation Genetics 11: 1405–1419.
19. Bearzi M (2005) Aspects of the ecology and behaviour of bottlenose dolphins (*Tursiops truncatus*) in Santa Monica Bay, California. Journal of Cetacean Research Management 7: 75–83.
20. Silva MA, Prieto R, Magalhaes S, Seabra M.I, Santos RS, Hammond PS (2008) Ranging patterns of bottlenose dolphins living in oceanic waters: implications for population structure. Marine Biology 156: 179–192.
21. Hersch SL, Duffield DA (1990) Distinguishing between Northwest Atlantic offshore and coastal bottlenose dolphins based on hemoglobin profile and morphometry. In: Leatherwood S, Reeves R, editors. The bottlenose dolphin. Academic Press California. pp. 129–139.
22. Perrin WF, Thieleking JL, Walker WA, Archer FI, Robertson KM (2011) Common bottlenose dolphins (*Tursiops truncatus*) in California waters: Cranial differentiation of coastal and offshore ecotypes. Marine Mammal Science 27: 769–792.
23. Kenney RD (1990). Bottlenose dolphins off the North Eastern United States. In: Leatherwood S, Reeves R, editors. The bottlenose dolphin. Academic Press San Diego, California. pp. 369–386.
24. Torres LP, Rosel PE, Read AJ, D'Agros C (2003) Improving management of overlapping bottlenose dolphin ecotypes through spatial analysis and genetics. Marine Mammal Science 19: 502–514.
25. Bearzi M, Saylan CA, Hwang A (2009) Ecology and comparison of coastal and offshore bottlenose dolphins (*Tursiops truncatus*) in California. Marine and Freshwater Research 60: 584–593.
26. Hoelzel AR, Potter CW, Best PB (1998) Genetic differentiation between parapatric 'nearshore' and 'offshore' populations of the bottlenose dolphin. Proceedings of the Royal Society of London Series B: Biological Sciences 265: 1177–1183. PMID: 9699311
27. Natoli A, Peddemors VM, Hoelzel AR (2004) Population structure and speciation in the genus *Tursiops* based on microsatellite and mitochondrial DNA analyses. Journal of Evolutionary Biology 17: 363–375. PMID: 15009270

28. Ingram SN (2000) The ecology and conservation of bottlenose dolphins in the Shannon estuary, Ireland. PhD thesis University College Cork, 213 p.
29. CODA: The Cetacean Offshore Distribution and Abundance in the European Atlantic (2008) Available online: http://biology.standrews.ac.uk/coda/documents/CODA_Final_Report_11-2-09.pdf. Accessed 20 April 2013.
30. O'Brien JM, Berrow SD, Ryan C, Mcgrath D, O'Connor I, et al. (2009) A note on long-distance matches of bottlenose dolphins (*Tursiops truncatus*) around the Irish coast using photo-identification. Journal of Cetacean Research and Management 11: 69–74.
31. Anderwald P, Haberlin MD, Coleman M, Ó Cadhla O, Englund A, Visser F, et al. (2012) Seasonal trends and spatial differences in marine mammal occurrence in Broadhaven Bay, North-West Ireland. Journal of the Marine Biological Association of the United Kingdom, 92: 1757–1766.
32. Robinson KP, O'Brien JM, Berrow SD, et al. (2012) Discrete or not so discrete: Long distance movements by coastal bottlenose dolphins in the UK and Ireland. Journal of Cetacean Research & Management 12: 365–372.
33. Irvine AB, Scott MD, Wells RS, Kaufmann JH (1981) Movements and activities of the Atlantic bottlenose dolphin, *Tursiops truncatus*, near Sarasota, Florida. Fisheries Bulletin 79: 671–688.
34. Würsig B, Würsig M (1977) The photographic determination of group size, composition, and stability of coastal porpoises (*Tursiops truncatus*). Science 198: 755–756.
35. Urian KW, Hofmann S, Wells RS, Read AJ (2009) Fine-scale population structure of bottlenose dolphins (*Tursiops truncatus*) in Tampa Bay, Florida. Marine Mammal Science 25: 619–638. doi: 10.1111/j.1748-7692.2009.00284.x
36. Wilson B, Hammond PS, Thompson PM (1999) Estimating size and assessing trends in a coastal bottlenose dolphin population. Ecological Applications 9: 288–300. PMID: 10401643
37. Scott MD, Wells RS, Irvine AB (1990) A long-term study of bottlenose dolphins on the West coast of Florida. In: Leatherwood S, Reeves R, editors. The bottlenose dolphin. Academic Press California. pp. 235–244.
38. Whitehead H (2008) Analyzing animal societies: quantitative methods for vertebrate social analysis. Chicago, University of Chicago Press. 336 p.
39. Whitehead H, Dufault S (1999) Techniques for analyzing vertebrate social structure using identified individuals: Review and recommendations. Advances in the Study of Behaviour 28: 33–74.
40. Cairns SJ, Schwager SJ (1987) A comparison of association indices. Animal Behaviour 35: 1454–1469.
41. Whitehead H (2009) SOCPROG programs: analyzing animal social structures. Behavioural Ecology and Sociobiology 63: 765–778.
42. Borgatti SP, Everett MG, Freeman LC (2002) UCInet for Windows: Software for Social Network Analysis. Harvard, MA: Analytic Technologies.
43. Department of Communication, Marine and Natural Resources Marine (2005) Notice No. 15 of 2005: Guidelines For Correct Procedures When Encountering Whales And Dolphins In Irish Coastal Waters. Available: http://www.dttas.ie/viewitem.asp?id=7936&lang=ENG&loc=2013. *Accessed 8 May 2013*.
44. Connor RC, Wells RS, Mann J, Read AJ (2000) The bottlenose dolphin: social relationships in a fission—fusion society. In: Mann J, Connor RC, Tyack PL, Whitehead H, editors. Cetacean societies: Field studies of dolphins and whales. Chicago: University of Chicago Press. pp. 91–126.
45. Defran RH, Weller D (1999) Occurrence, distribution, site fidelity, and school size of bottlenose dolphins (*Tursiops truncatus*) off San Diego, California. Marine Mammal Science 15: 366–380.
46. Defran RH, Weller DW, Kelly DL, Espinosa MA (1999) Range characteristics of Pacific coast Bottlenose dolphins (*Tursiops truncatus*) in the Southern Californian Bight. Marine Mammal Science 15: 381–393.
47. Baird RW, Mahaffy SD, Gorgone AM, Cullins T, McSweeney DJ, Oleson EM, et al. (2014) False killer whales and fisheries interactions in Hawaiian waters: Evidence for sex bias and variation among populations and social groups. Marine Mammal Science. doi: 10.1111/mms.12177
48. Baird RW, Gorgone AM (2005) False killer whale dorsal fin disfigurements as a possible indicator of long-line fishery interactions in Hawaiian waters. Pacific Science 59: 593–601.
49. Alves F, Quérouil S, Dinis A, Nicolau C, et al. (2013) Population structure of short-finned pilot whales in the oceanic archipelago of Madeira based on photo-identification and genetic analyses: implications for conservation. Aquatic Conservation Marine and Freshwater Ecosystems 23: 758–776.
50. Chu K, Nieukirk S (1988) Dorsal fin scars as indicators of age, sex, and social status in humpback whales (*Megaptera novaeangliae*). Canadian Journal of Zoology 66: 416–420.

51. Corkeron PJ, Morris RJ, Bryden MM (1987) Interactions between bottlenose dolphins and sharks in Moreton Bay, Queensland. Aquatic Mammal 13: 11–14.
52. Wells RJ, Scott MD (1997) Seasonal incidence of boat strikes on bottlenose dolphins near Sarasota, Florida. Marine Mammal Science 13: 475–480.
53. Luksenburg JA (2014) Prevalence of external injuries in small cetaceans in Aruban waters, Southern Caribbean. PLoS ONE 9(2): e88988. doi: 10.1371/journal.pone.0088988 PMID: 24586473
54. Heithaus MR (2001) Shark attacks on bottlenose dolphins (*Tursiops aduncus*) in Shark Bay, Western Australia: Attack rate, bite scar frequencies, and attack seasonality. Marine Mammal Science 17: 526–539.
55. Jaiteh VF, Allen SJ, Meeuwig JJ, Loneragan NR (2012) Subsurface behaviour of bottlenose dolphins (*Tursiops truncatus*) interacting with fish trawl nets in North Western Australia: Implications for bycatch mitigation. Marine Mammal Science 29: E266–E281. doi: 10.1111/j.1748-7692.2012.00620.x.
56. Allen SJ, Loneragan NR (2010) Reducing dolphin bycatch in the Pilbara Finfish Trawl Fishery. Final Report to the Fisheries Research and Development Corporation (FRDC). Murdoch University, Australia. 59 *p*. *ISBN*: 978-0-86905-926-5. *Available*: http://frdc.com.au/research/final-reports/Pages/2008-048-DLD.aspx. *Accessed 5 January 2015*.
57. Anon. (2009) Atlas of the Commercial fisheries around Ireland, Marine Institute, December 2009. 58 p. Available: http://oar.marine.ie/bitstream/10793/30/1/Atlas%20of%20the%20Commercial%20Fisheries%20Around%20Ireland%2009.pdf. Accessed *5 January 2015*.
58. Couperus AS (1997) Interactions between Dutch midwater trawl and Atlantic White-sided dolphins (*Lagenorhynchus acutus*) Southwest of Ireland. Journal of the Northwest Atlantic Fisheries Science 22: 209–218.
59. Rogan E, Mackey M (2007) Megafauna bycatch in drift nets for albacore tuna (*Thunnus alalunga*) in the NE Atlantic. Fisheries Research 86: 6–14.
60. Santos MB, Fernandez R, Lopez A, Martınez JA, Pierce GJ (2007) Variability in the diet of bottlenose dolphin, *Tursiops truncatus*, in Galician waters, North Western Spain, 1990–2005. Journal of the Marine Biological Association of the United Kingdom 87: 231–242.
61. Oudejans MG, Visser F (2010) First confirmed record of a living sei whale (*Balaenoptera borealis* (Lesson, 1828)) in Irish coastal waters. Irish Naturalists' Journal 31: 46–48.
62. Torres LP, Read AJ (2009) Where to catch a fish? The influence of foraging tactics on the ecology of bottlenose dolphins (*Tursiops truncatus*) in Florida Bay, Florida. Marine Mammal Science 25(4): 797–815.
63. Bejder L, Samuels A, Whitehead H, et al. (2006) Decline in relative abundance of bottlenose dolphins exposed to long-term disturbance. Conservation Biology 20: 1791–1798. PMID: 17181814
64. Bearzi G, Reeves RR, Notarbartolo-Di-Sciara G, Politi E, Ca adas A, Frantzis A, et al. (2003) Ecology, status and conservation of short-beaked common dolphins *Delphinus delphis* in the Mediterranean Sea. Mammal Review 33, No. 3, 224–252.
65. Read AJ, Drinker P, Northridge S (2006) Bycatch of marine mammals in U.S. and global fisheries. Conservation Biology 20: 163–169. PMID: 16909669
